# EHMTI-0290. Headaches in patients with autism spectrum disorder

**DOI:** 10.1186/1129-2377-15-S1-B37

**Published:** 2014-09-18

**Authors:** M Victorio

**Affiliations:** 1 NeuroDevelopmental Science Center, Akron Children's Hospital, Akron, OH, USA

## Introduction

Autism Spectrum Disorder (ASD) is a neurodevelopmental condition characterized by persistent deficits in social communication, social interaction and restricted, repetitive patterns of behavior, interests or activities. In general, ASD is associated with pain insensitivity and self-injurious behavior. Support for these associated traits are derived mostly from anecdotal and clinical observations. Headache disorders like migraine can be a disabling condition but none has been reported among individuals with ASD.

## Aims

To characterize the headache types experienced by patients with ASD and review their clinical profile.

## Methods

A retrospective chart review of patients with ASD who presented in the neurology clinic from January 2011 to April 2013 was performed.

## Results

Eighteen patients were identified, 12 males and 6 females. Migraine was the most frequent headache type occurring in up to 61% (11/18) of patients. Eight of these 11 patients have migraine without aura; one with migraine with aura and two patients have both migraine with and without aura. Combined migraine and tension type headache was seen in 3 patients. Three had chronic daily headache and one had probable migraine. Age at presentation ranged from 5-16 years. All patients were verbal and all have co-morbid behavioral and mental health conditions.

## Conclusion

Our data show that ASD patients, despite being known to have indifference to pain, can experience headaches; with migraine being the most common headache type in these patients referred in our neurology clinic.

No conflict of interest.

